# Spectral Properties of Single Gold Nanoparticles in Close Proximity to Biological Fluorophores Excited by 2-Photon Excitation

**DOI:** 10.1371/journal.pone.0124975

**Published:** 2015-04-24

**Authors:** Andrea Anzalone, Manuela Gabriel, Laura C. Estrada, Enrico Gratton

**Affiliations:** 1 Laboratory for Fluorescence Dynamics, Department of Biomedical Engineering, University of California Irvine, Irvine, California, United States of America; 2 Centre for Bioactive Discovery in Health and Ageing, School of Science & Technology, University of New England, Armidale, Australia; Universidad del Pais Vasco, SPAIN

## Abstract

Metallic nanoparticles (NPs) are able to modify the excitation and emission rates (plasmonic enhancement) of fluorescent molecules in their close proximity. In this work, we measured the emission spectra of 20 nm Gold Nanoparticles (AuNPs) fixed on a glass surface submerged in a solution of different fluorophores using a spectral camera and 2-photon excitation. While on the glass surface, we observed the presence in the emission at least 3 components: i) second harmonic signal (SHG), ii) a broad emission from AuNPS and iii) fluorescence arising from fluorophores nearby. When on the glass surface, we found that the 3 spectral components have different relative intensities when the incident direction of linear polarization was changed indicating different physical origins for these components. Then we measured by fluctuation correlation spectroscopy (FCS) the scattering and fluorescence signal of the particles alone and in a solution of 100 nM EGFP using the spectral camera or measuring the scattering and fluorescence from the particles. We observed occasional fluorescence bursts when in the suspension we added fluorescent proteins. The spectrum of these burst was devoid of the SHG and of the broad emission in contrast to the signal collected from the gold nanoparticles on the glass surface. Instead we found that the spectrum during the burst corresponded closely to the spectrum of the fluorescent protein. An additional control was obtained by measuring the cross-correlation between the reflection from the particles and the fluorescence arising from EGFP both excited at 488 nm. We found a very weak cross-correlation between the AuNPs and the fluorescence confirming that the burst originate from a few particles with a fluorescence signal.

## Introduction

**Fig 1:**
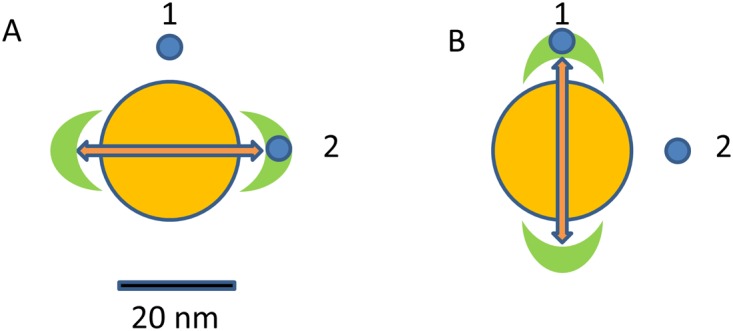
Schematic representation of selection of emission from a specific fluorophore in the proximity of the AuNP. The AuNP is shown schematically in yellow with regions of plasmonic enhancement in green. (A)In part A, the direction of linear polarization of the laser light is horizontal: particle 1 is not in the region of enhancement but particle 2 is. Therefore most of the fluorescence will arise from particle 2. (B)If the polarization of the laser beam is vertical, only the fluorescence from particle 1 will be enhanced.

Fluorescent molecules, either endogenous or exogenous are commonly used for dynamic imaging of cells and tissues [1, 2]. However fluorescent molecules are affected by photobleaching and quantum dots are affected by blinking making them undesirable for some microscopy applications [3] [4]. In recent years the use of metallic nanoparticles as fluorescent probes has grown exponentially. Metallic nanoparticles, in close proximity to fluorophores, could enhance the fluorophore excitation and emission rate. This effect is called plasmonic enhancement [5, 6]. The plasmonic enhancement is due to oscillations, induced by the light, of free electrons in a metal structure such as a gold nanoparticle (AuNP). Under some conditions which depend on the particle size, shape, the light wavelength and the refraction index of the medium, it is possible to have a surface resonance effect [7] which affects optical properties of fluorophores in close proximity to the metal nanoparticle. Properties affected include increasing the radiative decay rates of the molecule, enhancing the optical intensity incident and increasing the coupling efficiency of the fluorescence emission to the far field through nanoparticle scattering[7]. The emission from fluorophores in contact with the surface of metallic nanoparticle is quenched, while at the distance of few nanometers from the surface, the fluorescence could be strongly enhanced (plasmonic enhancement) [6]. Using 20 nm diameter AuNPs, the excitation energy is confined in a region extending up to about 10 nm near the surface of the AuNP where there is a strong enhancement of fluorescence when a fluorophore is in that location[8, 9]. The regions of enhancement depend on the orientation of linear light polarization as shown schematically in Fig 1.

Gold nanoparticles of different sizes have been used as labels for fluorescence microscopy [10, 11] however the spectral properties have not been determined at the single particle level. In this work we tracked individual AuNPs fixed on a glass surface using the orbital tracking method [12–14]. This technique allows to stay exactly on top of one particle for the relatively long duration of spectral acquisition, independently of the microscope stage or particle small movements [15]. The orbital tracking method coupled with 2-photon excitation near infrared microscopy has been previously used to follow the movement of gold nanoparticles along collagen fibers and actin filaments [10, 11]. The fluorescence enhancement obtained with strong pulses of near infrared radiation has been reported to be much larger than the enhancement obtained with continuous illumination using visible light [16]. Our long term goal is to track gold particles as they move in the nucleus of cells and to determine the emission of nearby fluorophores, revealing the presence of specific fluorophores in the enhancement region through their emission color. To develop this technology we need first to understand the spectral emission of single AuNPs under 2-photon excitation, which to our knowledge is not fully described and also the effect of changing the wavelength of excitation on the spectrum since we will like to detect different fluorophores simultaneously.

To advance in this direction, in this work we show the spectral signal of single AuNPs immobilized on a glass surface. Single AuNPs are excited at different light polarization angles and excitation wavelengths both in the presence and absence of EGFP and mCherry fluorescent proteins in the solution above the surface. We expect that the enhancement region for a given AuNP depends on the direction of incident polarization as schematically shown in Fig 1. Here we show that changing the orientation of polarization allows us to effectively explore different regions around the AuNP and to localize and distinguish fluorescent molecules in the proximity of the AuNP on the basis of their spectral signature. Since full spectral acquisition is relatively slow (about 200 μs per spectrum) we were able to measure the entire emission spectrum only for immobilized AuNPs or nanoparticles in viscous media. While on the surface, the observed spectrum of the gold nanoparticle could be due to specific defects/impurities of the surface or to specific properties of the gold nanoparticles. Since it is difficult to collect the spectrum of the gold nanoparticles excited by 2-photon excitation far from the surface and in the presence of fluorescent dyes in the solution, we resorted to fluctuation correlation spectroscopy (FCS) to obtain the emission spectrum of occasional bursts we observed during the FCS data acquisition.

## Results and Discussion

### Spectral analysis of fixed Gold Nanoparticles (AuNPs) with and without fluorophores in the media

**Fig 2:**
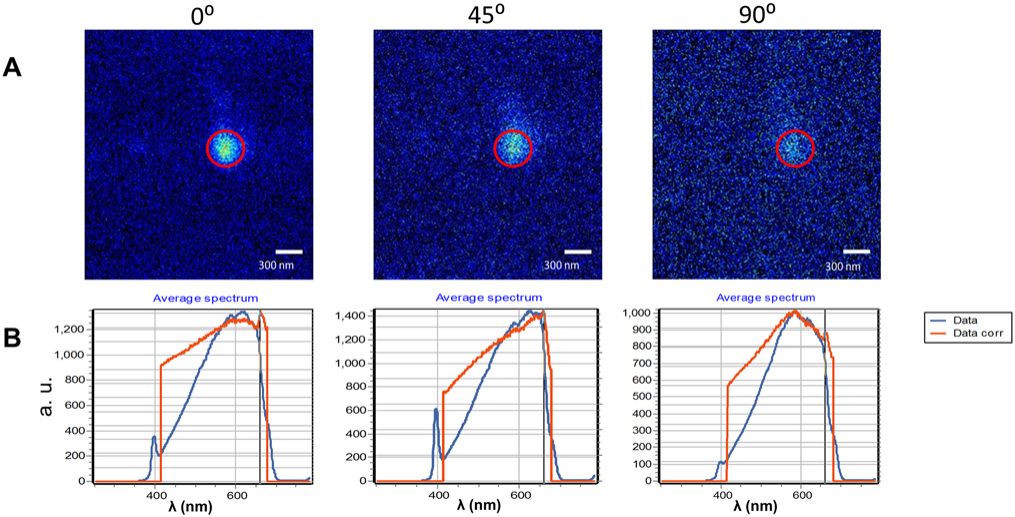
Raster scan images of a single gold nanoparticle fixed on a glass coverslip. (A)The sample is excited at 790 nm with 3 different orientations of linear polarized light. The emission is collected through a bandpass filter 520/30 nm. (B) Emission light collected with the spectral camera. The blue line in the spectrum is the raw data. The red line is the corrected intensity for the instrument response in the range 420–670 nm. Fig 2 shows 3 different polarization angles of excitation: 0°, 45° and 90°. The spectrum is characterized by a broad emission with a maximum around 600nm. The wavelength of the maximum is orientation dependent. There is a narrow feature at 395nm which is due to SHG.

In this section we describe the acquisition of emission spectra from 20 nm diameter AuNP nanoparticles fixed on a poly-lysine covered glass surface excited by 2-photon excitation with linearly polarized light. The particle position is first identified in a raster scan image using 2-photon excitation (Fig 2). Since the particle is fixed we could directly collect the spectrum from the pixel by focusing the laser on the particle. However, as we rotate the orientation of the polarizer, it is possible that the laser beam might be slightly displaced. Therefore we use the orbital tracking method to center the region of light collection exactly on the particle during the rotation of the polarizer. Another advantage of the orbital tracking method is that during the orbital motion (200nm radius) we explore a region around the AuNP. We set the spectral camera to acquire spectra along the orbit each 5.6° so that we can determine the angular distribution of the emission spectra from the AuNP along the orbit. Once the particle is centered, we use a calcite polarizer to rotate the orientation of the excitation light at 3 different angles, still maintaining the orbit exactly on the particle. The zero-orientation angle of the light polarization is defined with respect to the microscope as the direction of the x-scanning in the raster scan pattern. Following this definition, we collected spectra at an orientation of 0°, 45° and 90° of the polarizer. We used a Ti-Sapphire laser producing pulses of about 120 fs at 4 different excitation wavelengths, 740nm, 790nm, 840nm and 890nm. These wavelengths cover the 2-photon excitation range of known fluorescent biomolecules such as NADH and FAD endogenously present in cell and the excitation wavelengths of EEGFP and mCherry fluorescent proteins. The emission signal is analyzed subtracting the background measured in a position without AuNPs. We used a laser power in the range of 0.5 mW measured at the sample position. The laser power was kept constant while we rotated the polarizer angle. Using a solution of a fluorescent dye we verified that the rotation of the polarizer per se caused changes of less than 5% when the polarizer was moved the full circle. Fig 2A shows raster scan images of a single AuNP fixed on the glass surface excited at 790 nm. For the raster scan images, the emission signal is collected with a band pass filter at 520/30 nm to collect the emission of EEGFP and the polarizer is oriented at three different angles, 0°, 45° and 90°, respectively in the 3 panels of Fig 2A.

The emission spectrum of the AuNP at 3 different excitation polarization angles at 790 nm was acquired using the spectral camera (Fig 2B). Spectra in Fig 2 and in all figures of this paper are displayed using 2 lines, a blue line that represents the raw spectrum (after background subtraction) and a red line which is the corrected emission spectra. The calibration factors for the corrected spectrum were obtained using a calibrated tungsten lamp. The calibration with the tungsten lamp does not extend to the UV region (Fig 2B) so that corrected emission spectra are available only in the range 420nm-670 nm. However narrow spectral features outside the calibration range such as SHG are still visible but their intensity is not calibrated. The orientation of the excitation polarization affects the overall integrated emission collected from the AuNP excited at 790 nm (Fig 2B) as well as at other excitation wavelengths (S1 Fig). The effect of changing the polarizer angle is unexpected given the spherical symmetry of the AuNP. The AuNP emission occurs over a wide spectral region, previously reported for metallic particles (Fig 2B) [17]. We note that the narrow feature at 395nm (Fig 2), corresponding to second harmonic generation, has larger relative emission at 45° polarization direction.

**Fig 3:**
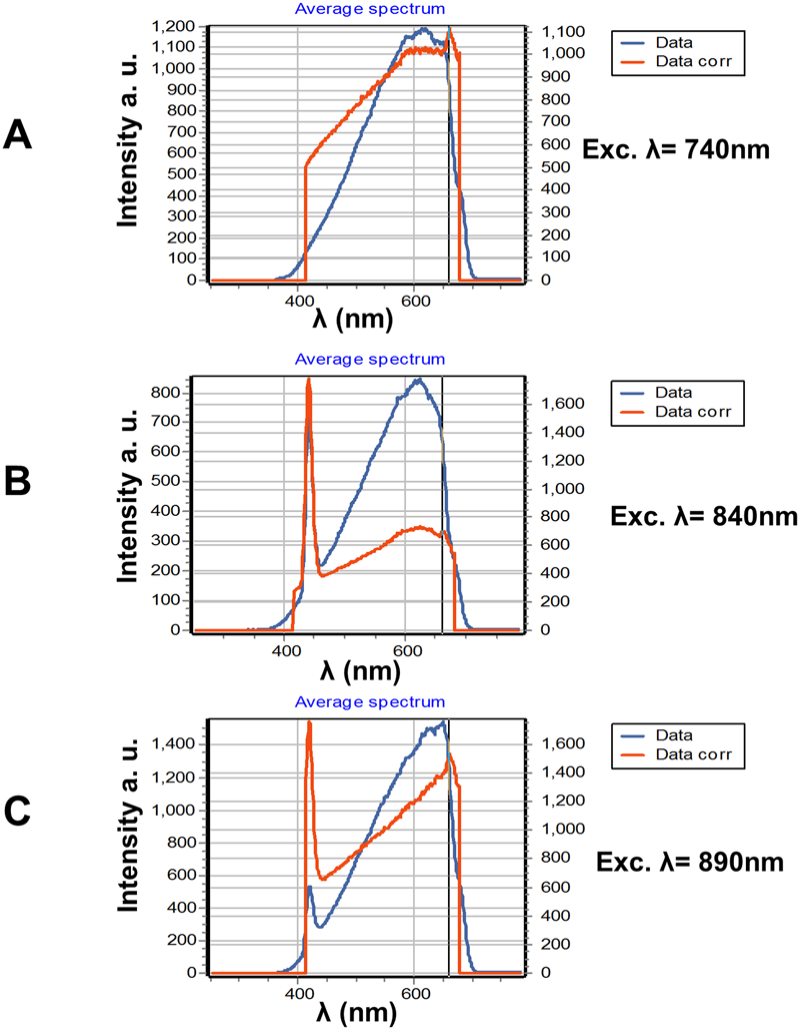
Emission spectrum of the same AuNP of Fig 2 with a polarization angle of 45° excitation wavelength of (A) 740 nm, (B) 840nm and (C) 890nm. In C and B the SHG signal is very strong. The broad emission spectrum maximum depends in the wavelength of excitation.

**Fig 4:**
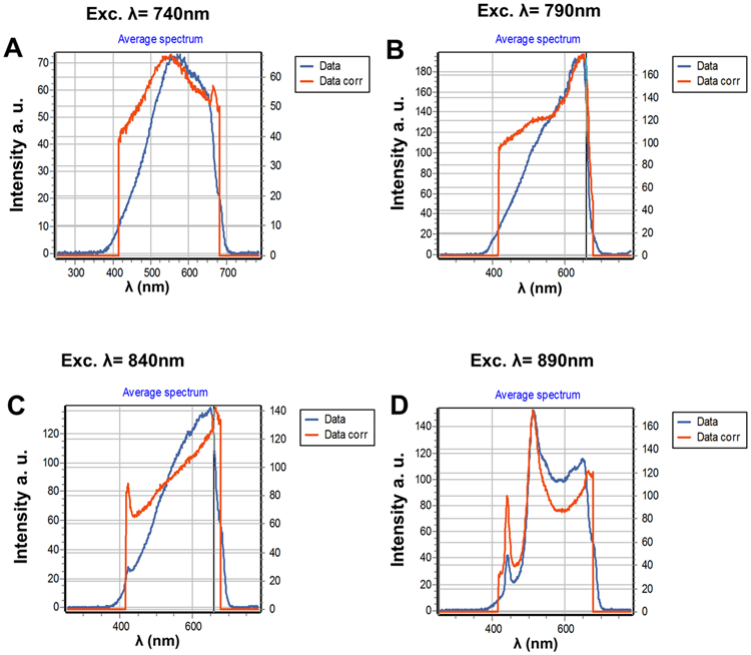
Spectrum of a AuNP excited at 4 different wavelengths and with an orientation of 45 degrees for the linear polarizer. **This sample was added with a solution of 100nM EGFP**. (A) EGFP is minimally excited at 740 nm and we see the typical spectrum of the metallic nanoparticle. (B) at 790nm we broad emission of the nanoparticle shifts toward the red and a band starts to appear in the 520nm region typical of EGFP. (C) at 840nm the SHG signal starts to be observed. (D) at 890 nm the fluorescence from the EGFP is strongly enhanced and this fluorescence is clearly distinguishable in the 529 nm region. At this excitation wavelength there is also a very strong SHG signal.

**Fig 5:**
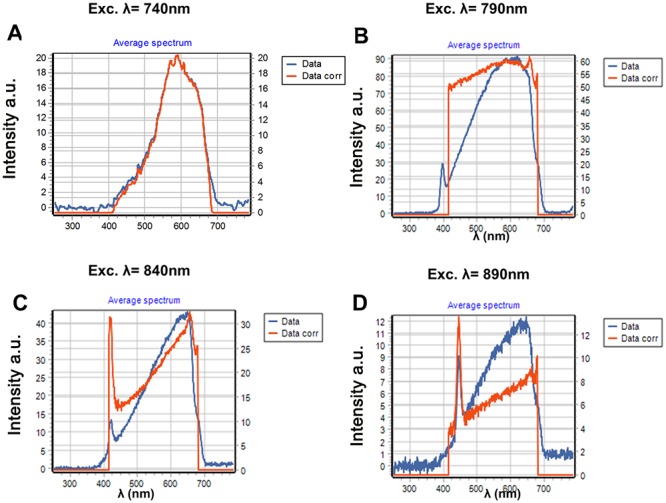
Spectrum of a AuNP excited at 4 different wavelengths and with an orientation of 45 degrees for the linear polarizer. This sample was added with a solution of 100nM mCherry. (A) mCherry is excited at 740 nm and we see the typical spectrum of the mCherry. The contribution of mCherry is not distinguishable at other excitation wavelengths. (B) At 790nm we observe the broad emission of the nanoparticle. (C) At 840nm the SHG signal starts to be observed. (D) At 890 nm the fluorescence mCherry is not excited and we can only see a strong SHG in addition to the broad fluorescence from the nanoparticle.

**Fig 6:**
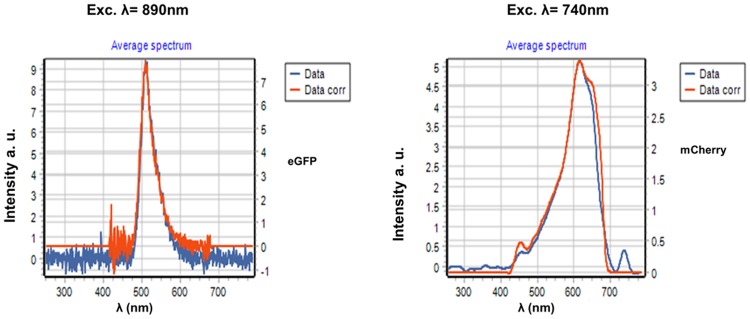
Emission spectrum of EGFP and mCherry in a region of the sample with no AuNP. The excitation wavelengths were chosen to maximally excite each of the fluorophores, 890nm and 740nm, respectively.

In Fig 3A we show the spectra of the same AuNP fixed on the glass excited at 740, 840 and 890 nm using 45 degrees for the orientation of the linear polarization. At 740 nm excitation we observe spectra comparable with the spectra of Fig 2, i.e., a broad spectrum with an intensity comparable to the one recorded at 790 nm (Fig 2B). When the sample is excited at 840 nm and 890 nm, we observe a strong second harmonic generation signal (SHG) (Fig 3B and 3C). The SHG is not visible when we excite at 740 nm or 790 nm, due to the camera cutoff at about 420 nm that prevents observation of SHG at the lower excitation wavelengths. The peak count of the SHG is higher than that of the broad emission (Fig 3B and 3C). To exclude the possibility that impurities on the glass alone were generating the SHG we measured the spectra centering the orbit in an area without AuNPs. The glass alone does not show any SHG or fluorescence emission under our illumination conditions. We then added on top of the slide a solution of 100 nM of purified EGFP or mCherry, respectively (Figs 4 and 5). Figs 4 and 5 show spectral characteristics of the AuNP alone plus a feature that depends on the protein added. We excited the samples at 4 wavelengths, 740 nm, 790 nm, 840 nm and 890 nm and with 3 different polarization angles; however for brevity we only report data for 4 excitation wavelengths and 45° orientation of polarization. When we excite at 840 nm and 890 nm we observe SHG signal comparable to the one observed in the absence of the fluorophores. At 890 nm excitation we can clearly identify a peak of emission corresponding to EGFP (Fig 4D) in addition to the spectrum of the AuNP. Fig 5, shows the spectra when 100 nM mCherry was added to the sample. We could clearly see the spectrum of mCherry at 740nm excitation (Fig 5A). At all other wavelengths mCherry is weakly or not excited and at these wavelengths we can see the broad spectrum and the SHG signal. In Fig 6 we show the spectra of EGFP and mCherry acquired on the same sample but away from the AuNP. The intensity recorded using the same laser power in a region of the same sample without AuNP, was very weak (9 counts at the maximum when EGFP was excited at 890nm and 5 counts when Cherry was excited at 740 nm).

**Fig 7:**
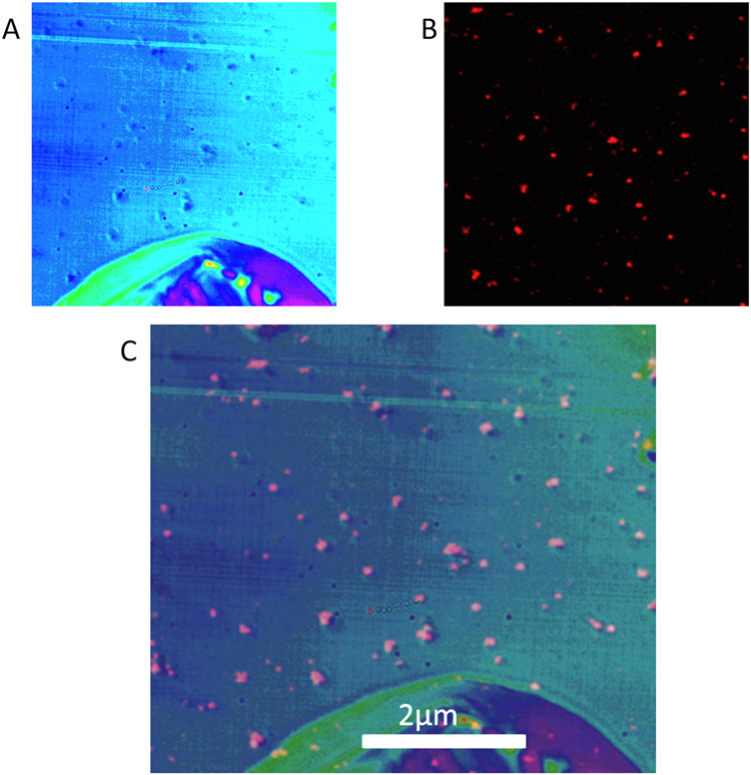
DIC image of AUNPs. (A) DIC image. The large structure on the bottom is an impurity over the glass surface that was used to guide the recognition of the same area for the fluorescence image. (B) Fluorescence image of a small region of the slide with fixed AuNPs excited at 890 nm with emission band pass filter 560–650 nm. The image in B is strongly contrasted to make the AuNP visible. A threshold of 20 levels above the background was applied to the fluorescence image to select only the fluorescent AuNPs. (C) Overlay of the DIC and fluorescence image. All particles in B have a corresponding DIC signal in A, but not all dots in A have a corresponding fluorescence dot in B.

The conclusion of the measurements of the spectra of AuNP immobilized on a glass surface is that AuNP has a very strong signal with a spectrum characterized by SHG and a broad emission. The overall emission and the relative amount of SHG depended on the polarizer orientation. Given the spherical symmetry of the nanoparticle this observation was not expected. We summarized our observations as follows:

1. Different AuNPs show similar behaviors with respect to the overall appearance of SHG and broad emission spectrum but the polarization orientation at which the maximal signal is obtained depends on the specific AuNP selected. This result implies that it is the lack of symmetry of the environment of the AuNP that causes the signal to be maximal at a specific orientation of the polarizer. We also noticed that when we measured the spectrum along the orbit during tracking, the SHG and the broad emission spectrum appeared displaced by 90° (S2 Fig). Our interpretation is that the broad emission as well as the SHG originates from impurities/defects of the glass surface. These defects could be located randomly around a given AuNP. We also performed experiments with immobilized fluorescent beads and we found no polarization effects for the beads (S3 Fig).
2. When we added a specific fluorophore (EGFP and mCherry) we observed an additional signal which has the same spectrum of the fluorophore alone, but the intensity of the spectrum measured in close proximity to the AuNP is strongly enhanced. Also the spectrum of the dye is larger at specific angles along the orbit and the position of the maximum signal depends on the polarizer orientation. We believe that dye molecules (one or more) are found at specific locations in the region of fluorescence enhancement of the AuNP and that they are immobilized.
3. The emission of single AuNP shows that the spectrum is composed by at least three contributions: i) SHG that can be maximized depending on the excitation wavelength and polarization, ii) a broad emission that seems to be characteristic of the AuNP itself when on a surface and iii) a component that could exists or not which is due to the proximity of the AuNP to a dye molecule and/or defects of the glass surface. Also the dye signal appears at specific orientations of light polarization.
4. We obtained DIC (differential interference contrast) images with a lamp and a camera as well as fluorescence images excited with 2-photon excitation at 890nm of the same area. In Fig 7 we show that in the area examined, only a fraction of what appear to be AuNPs on the glass surface show fluorescence. This experiment shows that not all AuNPs emit fluorescence and SHG, which corroborate our hypothesis that the measured broad emission and SHG is due to defects/impurities of the glass surface. In the absence of AuNPs we cannot find the DIC small dots signals characteristic of the AuNPs.

### Spectral analysis of AuNPs in a suspension away from the glass surface with and without fluorophores in the media

**Fig 8:**
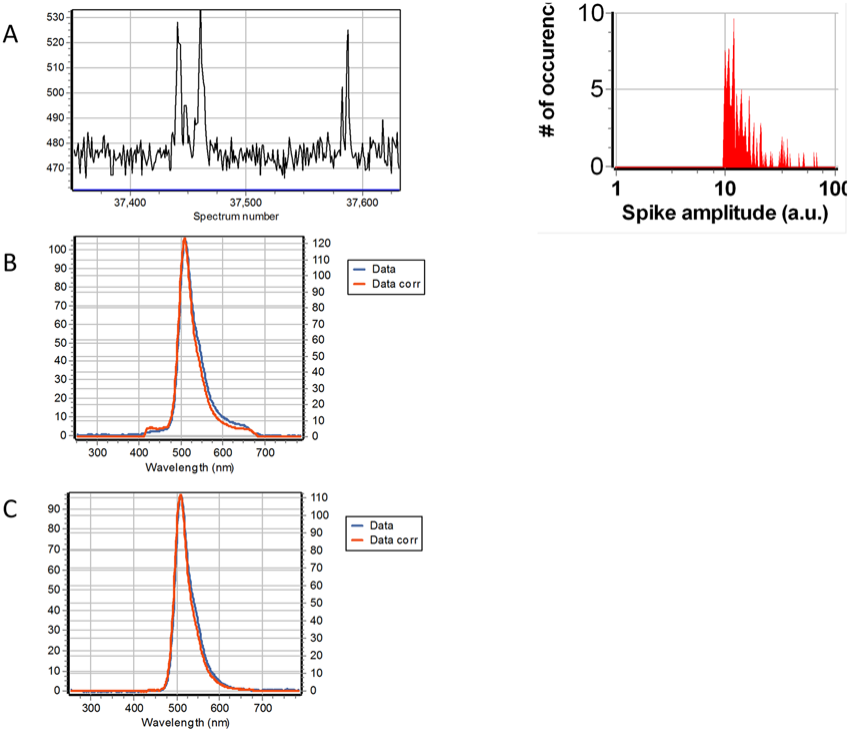
1 nM AuNP suspension in the presence of 100nM EGFP. The excitation wavelength was 890 nm. In all graph the vertical axis is in intensity units from the Andor camera. (A) Intensity trace of a small region of the overall data collection. The intensity is obtained by the integrating the spectrum from 420nm to 680nm. A total of 500,000 spectra were collected every 200 microseconds. Occasional burst are observed along the intensity trace above the average spectral intensity due to EGFP in solution. (B) The average spectrum of 144 intensity bursts. (C) Average spectrum of the regions of the trace without bursts. The spectra in B and C are very similar.

**Fig 9:**
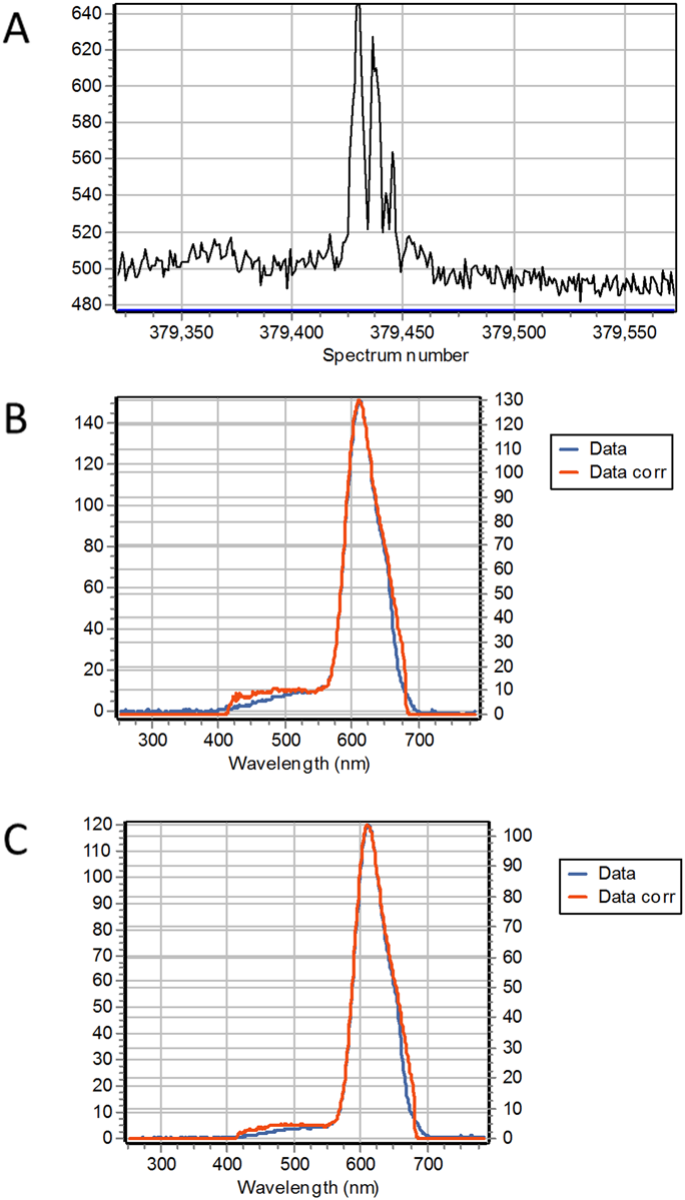
1 nM AuNP suspension in the presence of 100nM mCherry. The excitation wavelength was 740 nm. In all graph the vertical axis is in intensity units from the Andor camera. (A) Intensity trace of a small region of the overall data collection. The intensity is obtained by the integrating the spectrum from 420nm to 680nm. A total of 500,000 spectra were collected every 200 microseconds. Occasional burst are observed along the intensity trace above the average spectral intensity due to mCherry in solution. (B) The average spectrum of 80 intensity bursts. (C) Average spectrum of the regions of the trace without bursts. The spectra in B and C are very similar.

**Fig 10:**
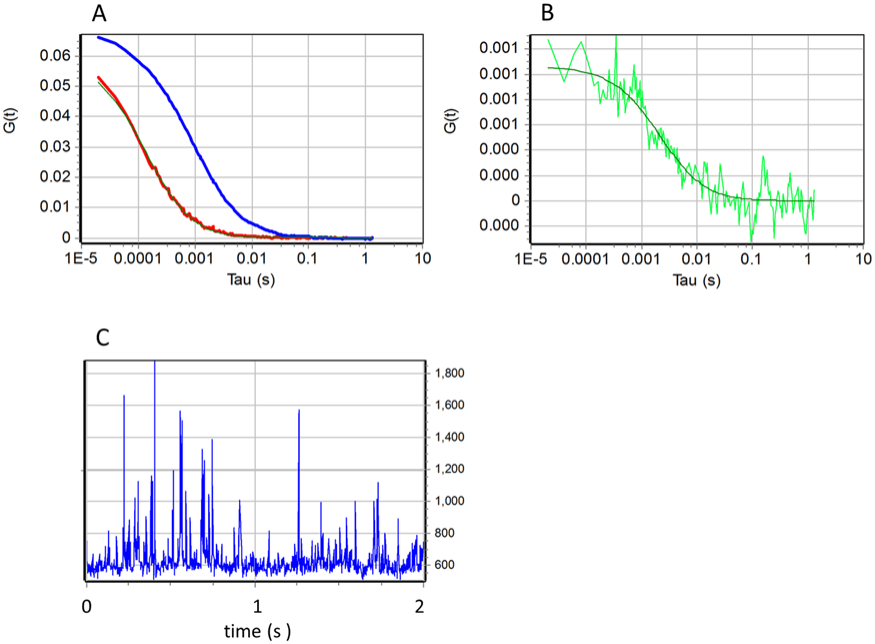
Solution fluctuation spectroscopy of 1 nM AuNP nanoparticles and 100nM EGFP. (A) Autocorrelation function of 1nM suspension of gold nanoparticles using a laser at 488nm and detecting the particle in confocal reflection (blue line) and of 100 nM EFGP excited at 488nm and viewed with an emission bandpass at 525/20 (red curve). When the AuNPs and the EGFP where together in the same sample the two autocorrelation curves remain almost identical. (B) Cross-correlation between the two channels (green curve) and the fit (black) using the same parameters for the fit of the autocorrelation curve of the sample with the AuNPs alone. (C) Intensity fluctuations in the reflection channel showing spikes due to the AuNPs passing in the volume of excitation.

Since part of the signal from immobilized AuNPs could originate from surface defects, we performed FCS experiments in a suspension of AuNPs in 50% glycerol in the presence and absence of fluorescent proteins. We use a suspension of 1nM AuNP in the presence of 100nM EGFP (Fig 8) and also 100nM mCherry (Fig 9). The FCS trace is characterized by few very intense fluorescence bursts. Figs 8A and 9A shows a small region of the FCS trace where a burst is detected. The spectrum during the burst (Figs 8B and 9B) is identical to the spectrum in the region of the trace away from the burst (Figs 8C and 9C). If the AuNP had a fluorescent emission per se at the specific wavelengths of excitation employed, we should have seen a very large signal with the characteristic putative spectrum of the AuNPs. Given the particle concentration (1nM) and the excitation volume of the 2-photon excitation (about 0.2 fL) we should excite on the order of 1 particle on the average at any instant of time. Clearly, we don’t see any emission from the gold *per se*, instead we observe few rare intensity bursts. If the excitation volume for these burst is determined by the enhancement region around the AuNPs, the effective volume of excitation for the fluorescence should be much smaller, on the order of the 1000nm^3^ per particle. In this volume we should have about 1 particle on the average which could explain the presence of occasional bursts (much less than one per second at 1nM AuNP and 100nM fluorescent protein). As a control we measured the fluctuations without particles but with the EGFP and the fluctuations of the particle suspension without the EGFP. In each case the bursts were absent. In a separate experiment we measure the fluctuations of a suspension of AuNPs in buffer in presence and absence of 100nM EGFP. In this experiment we used excitation at 488nm form a CW argon ion laser. To detect the AuNPs we use confocal reflection and at the same time we could record the intensity in the fluorescence channel centered at 515/20 nm. Fig 10 shows the autocorrelation curve for the sample with the gold alone and for a sample of 100nM EGFP. With the gold alone, the intensity in the fluorescence channel was negligible. The analysis of the ACF gives a diffusion coefficient for the 20 nm AuNP of 12.8 μm^2^/s. The 100nM sample gives an autocorrelation that can be fitted with a single component with a diffusion coefficient of 88.66 μm^2^/s. For the sample in which we have both the AuNPs and 100nM EGFP we found a very weak cross-correlation indicative of very few events in which the AuNPs show some fluorescence. This relatively small cross-correlation signal is consistent with the spectral information in Figs 8 and 9 where we measured occasional fluorescence burst when AuNPs were in the presence of the fluorescent proteins.

## Conclusions

Emission from AuNPs fixed on a glass surface is characterized by a broad spectrum and a narrow component that can be assigned to SHG. AuNPs in close proximity to fluorescent molecules are able to increase the excitation and emission rate of different dyes emitting at different colors. On the basis of the excitation at different polarization orientations, for AuNP immobilized on a glass surface we can show that these emissions features appear at different location on the glass in the proximity of the AuNP, probably from the regions of enhancement since they appear twice as we rotate the polarization orientation by full 360 degrees. In a 1nM AuNP suspension focusing away from the glass surface, we found no evidence of emission from the AuNPs. In a suspension of 1nM AuNP and 100 nM fluorescent proteins such as EGFP and mCherry we observed few bursts when we focused away from the surface. The analysis of the spectrum of the bursts shows that the emission is identical to the emission of the fluorescent protein in the regions without bursts. Our experiments are significant since they prove that we can detect the position and the emission spectra of fixed fluorophores. In the context of using AuNPs in cells, we could detect and precisely localize the emission from relatively fixed fluorophores using spectral detection and using the 3D orbital tracking techniques to determine the exact position of the AuNP. By changing the polarization angle while tracking we could determine the angular position of the emission with respect to the center of the orbit. In the cellular environment we could use in the emission path, filters with emission bandpass tuned to specific fluorophores so that acquisition could be much faster than the collection of the entire emission spectrum shown in this paper.

## Materials and Methods

### Spectral Camera acquisition and sample preparation

For the spectral analysis we used a modified Olympus FV1000 (Olympus, Japan) microscope coupled to a 512-channel ultrafast EMCCD (Andor iXon Ultra). Briefly, the NIR beam from a MaiTai HP Ti:Sapphire laser is coupled into a commercial inverted Olympus FV1000 confocal system via the near-IR port. The laser power is controlled by an acoustic optical modulator. A RDM690 excitation dichroic mirror reflects the excitation beam into the scanning path and allows the collected signal passing through to the detectors in the de-scanned path. A 40× water objective (UPlanSApo, NA 1.2) was used for these measurements. The emission signal is directed to the spectrograph (Andor SR303i) equipped with the ultrafast EMCCD (Andor iXon Ultra). Typical acquisition time for one spectrum is 200 microseconds. The data set (100,000 to 500,000 spectra) is imported and analyzed using SimFCS software (http://www.lfd.uci.edu/). The EMCCD camera is synchronized with the pixel clock for spectra acquisition [12]. This approach allows also triggering the camera during the circular scanning giving spectral data 360° around the object which is at the center of the orbital motion. The orientation of the linear polarization of the excitation light was changed using a calcite polarizer (Thorlabs, USA) and a combination of quarter wave plates to produce a circularly polarized light before the polarizer. The AuNPs were fixed on MatTek 35 mm plates with glass bottom for optical imaging. The bottom glass was coated with 1% poly-lysine (Sigma-Aldrich). The purified proteins mCherry and EGFP are commercially available from Biovision Inc, USA.

### Microscope setup for the orbital tracking

The orbital tracking technique is implemented by 2 galvanometer-motor actuated mirrors to produce the circular orbit. The fluorescence light from the sample is collected using a 60X objective NA1.2 water objective (Olympus, Tokyo Japan). The instrument is controlled and data analyzed using the SimFCS software (http://www.lfd.uci.edu/). The orbital tracking method has already been described in previous work [10, 11, 15]. Briefly, in the orbital tracking method the laser beam traces circular orbits around the particle above and below. When the particle is at the center of the orbit, the fluorescence intensity is constant as the laser beam performs the orbits. We synchronized the circular motion with the camera spectral acquisition at specific angles along the orbit, for example each 4 degrees. For spectral acquisition the particle is fixed, the orbit (200nm diameter) is exactly centered on the particle by the feedback tracking algorithm.

### Microscope setup for the spectral FCS

The microscope setup is the same as described above for the orbital tracking. For the FCS measurements, the laser beam is focused in the suspension far away from the glass surface. The beam is stationary and the camera is triggered every 200 microseconds for a total collection of 500,000 spectra in 100 seconds. The trace intensity is obtained by integrating the emission spectrum from 420 nm to 680nm. The intensity bursts are selected using the routine “Correlation filter” of the SimFCS software.

### Sample preparation

20 nm gold nanoparticles (AuNP) were obtained from Sigma, USA. The fluorescent proteins (EGFP, mCherry) were obtained from Biovision Inc. (USA). The sample were prepared in a 50% glycerol aqueous solution where the AuNPs were added to obtain a final concentration of 1nM in the presence of 100 nM of EGFP or mCherry, respectively.

## Supporting Information

S1 Fig 20 nm Gold nanoparticle excited at 740 nm. Emission light collected with the spectral camera. The blue line in the spectrum is the raw data. The red line is the corrected intensity for the instrument response in the range 420–670 nm. S1 fig shows 3 different polarization angles of excitation: 0°, 45° and 90°. The spectrum is characterized by a broad emission with a maximum around 560–600nm. The wavelength of the maximum is orientation dependent.
(TIF)

S2 Fig Fixed AuNP excited at 880nm. A) The average spectrum at all polarization angles. B) The fraction of the spectrum in the SHG region at 440 nm (red area) is larger in the quadrant 0–90 and 180–270 degrees with respect to the emission in the region between 500 to 650nm (blue area) which is larger in the region between 90–180 and 270–360 degrees.
(TIF)

S3 Fig Raster scan images of a single 200 nm beads fixed on a glass coverslip. The sample is excited at 790 nm with 3 different orientations of linear polarized light. The emission is collected through a bandpass filter 520/30 nm.
(TIF)
