# Supplementary figures and images for: Spectral Properties of Single Gold Nanoparticles in Close Proximity to Biological Fluorophores Excited by 2-Photon Excitation
Source: PLoS One. 2015 Apr 24;10(4):e0124975. doi: 10.1371/journal.pone.0124975 (PMC4409109; doi:10.1371/journal.pone.0124975)

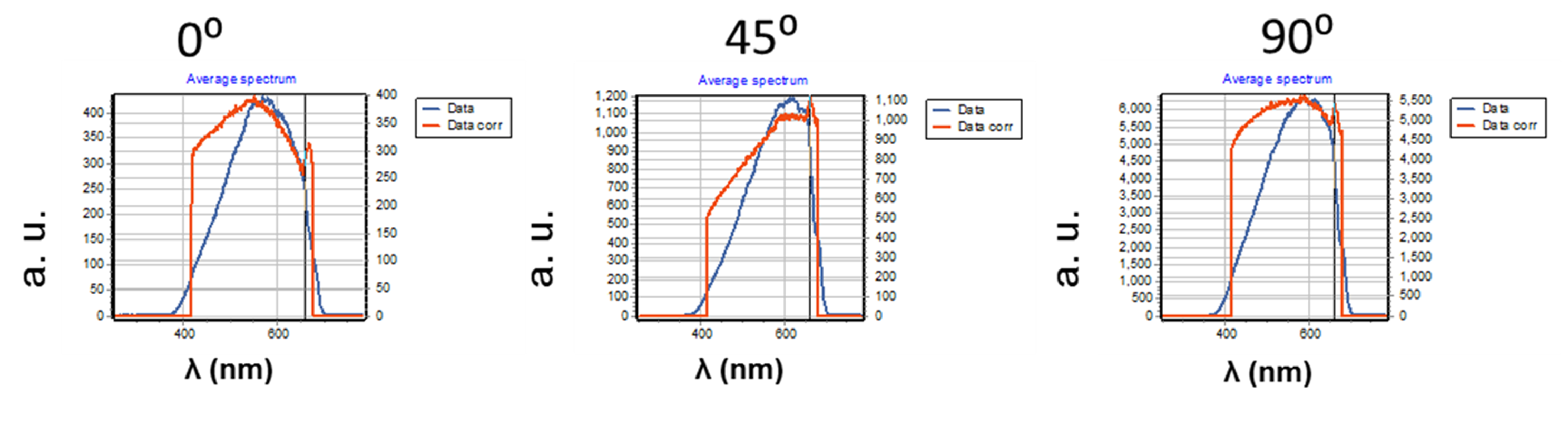

Supplement: S1 Fig — Emission light collected with the spectral camera. The blue line in the spectrum is the raw data. The red line is the corrected intensity for the instrument response in the range 420–670 nm. S1 fig shows 3 different polarization angles of excitation: 0°, 45° and 90°. The spectrum is characterized by a broad emission with a maximum around 560–600nm. The wavelength of the maximum is orientation dependent. (TIF) [file pone.0124975.s001.tif]

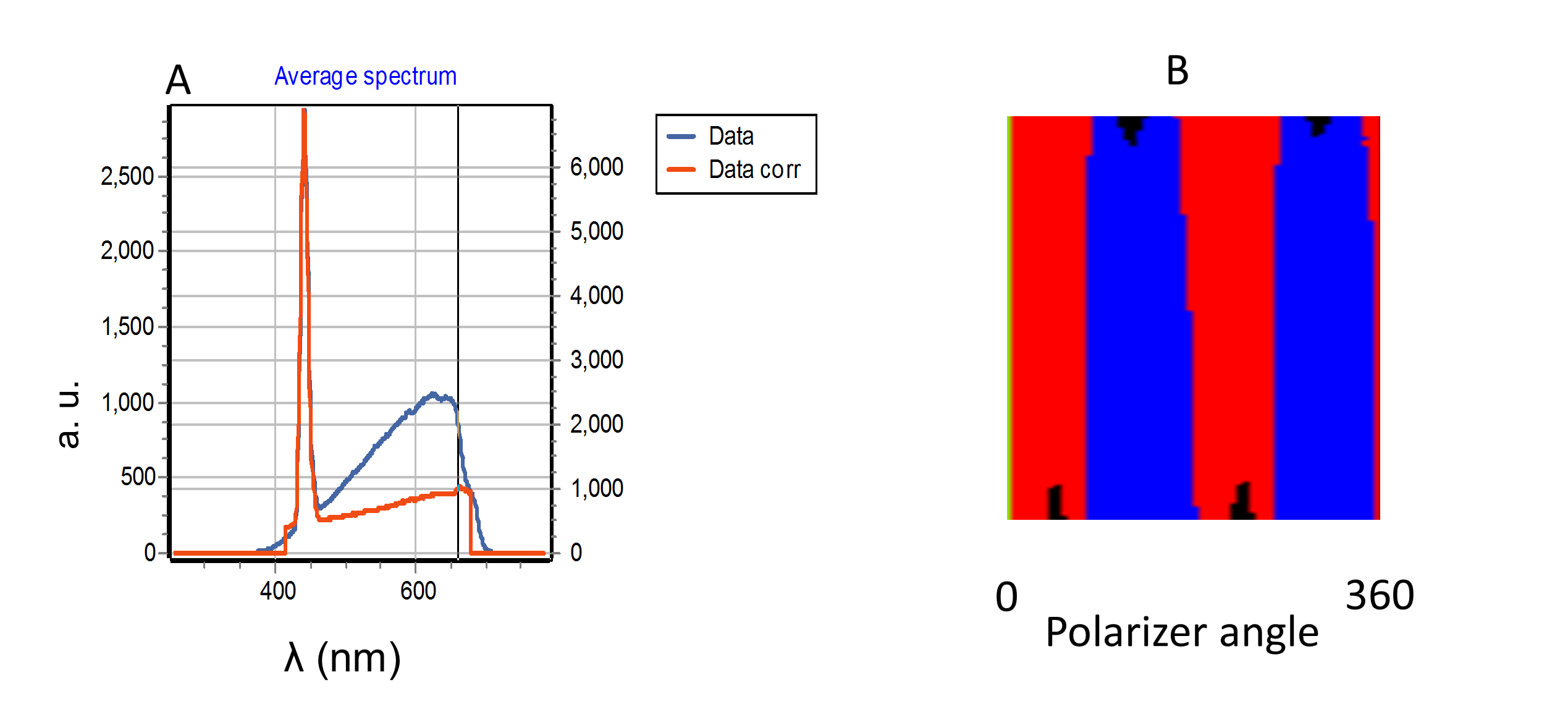

Supplement: S2 Fig — A) The average spectrum at all polarization angles. B) The fraction of the spectrum in the SHG region at 440 nm (red area) is larger in the quadrant 0–90 and 180–270 degrees with respect to the emission in the region between 500 to 650nm (blue area) which is larger in the region between 90–180 and 270–360 degrees. (TIF) [file pone.0124975.s002.tif]

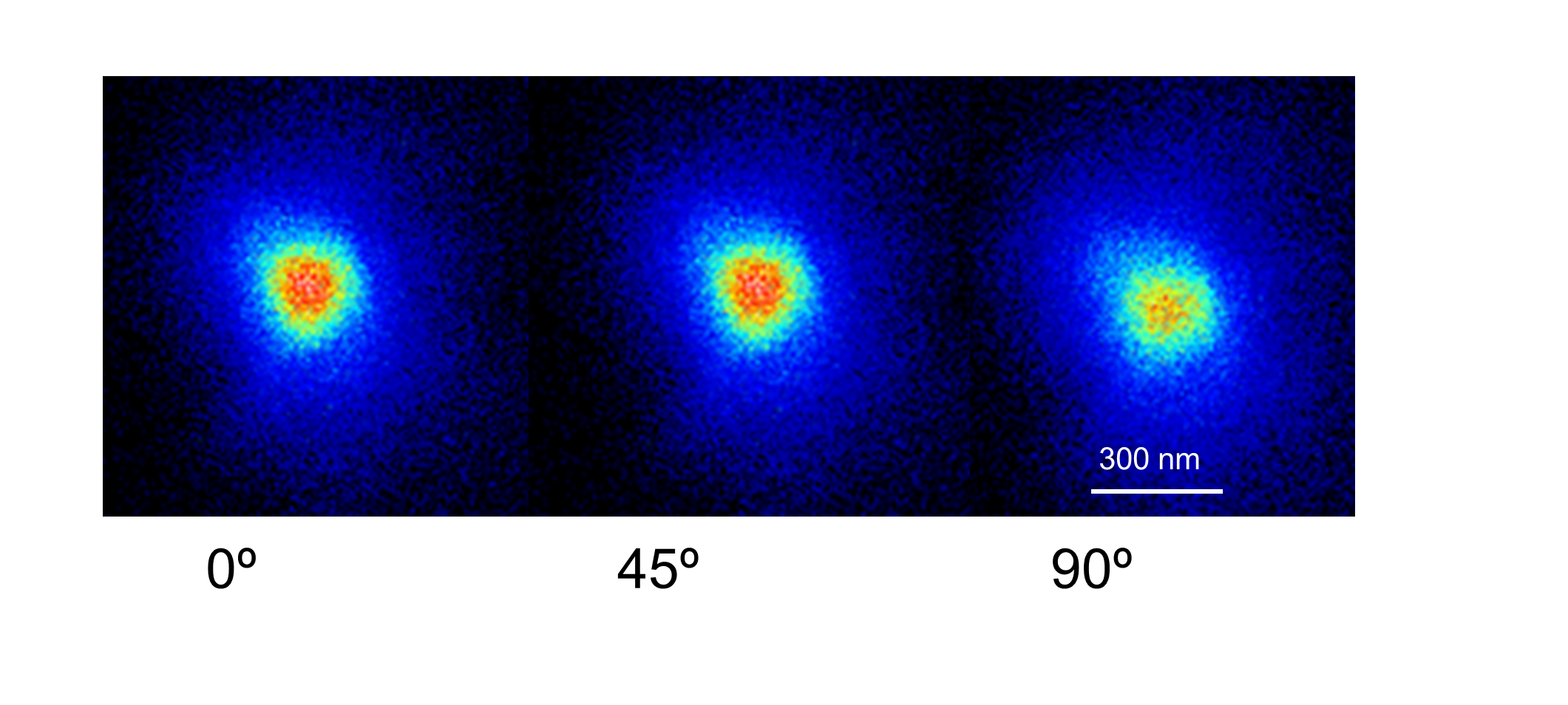

Supplement: S3 Fig — The sample is excited at 790 nm with 3 different orientations of linear polarized light. The emission is collected through a bandpass filter 520/30 nm. (TIF) [file pone.0124975.s003.tif]
